# Supplementary material for: Holothurin A Inhibits RUNX1-Enhanced EMT in Metastasis Prostate Cancer via the Akt/JNK and P38 MAPK Signaling Pathway
Source: Mar Drugs. 2023 Jun 3;21(6):345. doi: 10.3390/md21060345 (PMC10301049; doi:10.3390/md21060345)
Supplement: Supplementary file 1 [file marinedrugs-21-00345-s001.zip › marinedrugs-2417058-supplementary.pdf]

Supplementary figures

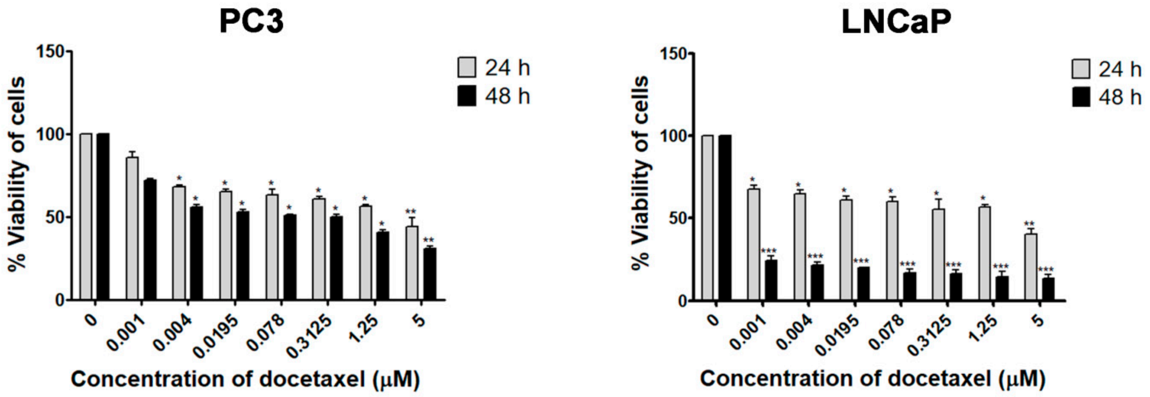

Supplementary Figure S1. Docetaxel treatment affects the viability of human PCa cell lines (PC3 and LNCaP). PCa cell lines were exposed to various concentrations of docetaxel at 24 and 48 h. MTT assay was performed to evaluate the cell viability. Values represent mean ± SD. *p<0.05; **p<0.01; ***p<0.001. The IC_{50} of docetaxel in PC3 were 2.01 and 0.45 µM, whereas for LNCaP were 2.20 and 0.001 µM, respectively.

Supplementary Table

Supplementary Table S1. The primers used for Real-time qPCR.

Gene	Forward primer	Reverse primer
GAPDH	5′-GAAAGCCTGCCGGTGACTAA-3′	5′-GCATCACCCGGAGGAGAAAT -3′
RUNX1	5′-AGGCAGGACGAATCACTG-3′	5′-AGTGTGATTCGTCCTGCCTG-3′
MMP 2	5′-TTGACGGTAAGGACGGACT-3′	5′-CTTGCAGTACTCCCCATCG-3′
MMP 9	5′-TTGACAGCGACAAGAAGTGG-3′	5′-CCCTCAGTGAAGCGGTACAT-3′
E-cadherin	5′-CGGGAATGCAGTTGAGGATC-3′	5′-AGGATGGTGTAAGCGATGGC-3′
Vimentin	5′-GCAGAATCATCACGAAGTGG-3′	5′-GCATGGTGATGTTGGACTCC-3′
